# Serum neuron specific enolase – impact of storage and measuring method

**DOI:** 10.1186/1756-0500-7-726

**Published:** 2014-10-15

**Authors:** Malin Rundgren, Tobias Cronberg, Hans Friberg, Anders Isaksson

**Affiliations:** Department of Clinical Sciences, Division of Anaesthesia and Intensive Care, Lund University, Lund, Sweden; Department of Clinical Sciences, Division of Neurology, Lund University, Lund, Sweden; Department of Laboratory Medicine, Division of Clinical Chemistry and Pharmacology, Lund University, Lund, Sweden; Department of Intensive and Perioperative Care, Skane University Hospital, Lund, 221 85 Lund, Sweden; Center For Resuscitation Science, Skane University Hospital, Lund, Sweden

**Keywords:** Biomarkers, Cardiac arrest, Hypothermia, Methodology, Neuron specific enolase, NSE, Prognostication

## Abstract

**Background:**

Neuron specific enolase (NSE) is a recognized biomarker for assessment of neurological outcome after cardiac arrest, but its reliability has been questioned. Our aim was to investigate what influence storage of samples and choice of measuring methods may have on levels of NSE in peripheral blood.

**Methods:**

Two serum samples were drawn simultaneously from 51 hypothermia treated cardiac arrest patients. One sample (original sample) was analysed when collected, using the Diasorin-method (LIAISON®NSE, LNSE). The other sample was frozen, stored at −70°C (stored sample), and reanalysed in the same laboratory 4–7 years later using both the Diasorin method and a Roche-method (NSE Cobas e601, CNSE). In addition, a comparison of the two methods was performed on 29 fresh samples.

**Results:**

The paired NSE results in original and stored samples were not significantly different, using the LNSE-method. The two methods produced significantly different results (p < 0.0001) on the paired, stored samples, with the CNSE method yielding higher values than the LNSE-method in 96% of samples. The CNSE method resulted in 36% higher values on average. In the method comparison on fresh samples, the CNSE-method generated on average 15% higher values compared to the LNSE-method, and the difference between the paired results was significant (p < 0.0001).

**Conclusion:**

The CNSE method generated consistently higher NSE-values than the LNSE method and this difference was more pronounced when frozen samples were analysed. Tolerability for prolonged freezing was acceptable.

## Background

Neuron specific enolase (NSE) is a dimeric intracellular glycolytic enzyme, comprised of two subunits, γγ or αγ. It is present in neurons and in other cells of neuroectodermal origin, but is also found in erythrocytes [1] and platelets [2]. Its half-life in serum is estimated to be 30 h [3]. Haemolysis produces an increase in NSE in serum in proportion to the degree of haemolysis [1, 3].

Serum NSE is the only biochemical marker of brain injury that has been incorporated into guidelines for neurological prognostication after cardiac arrest [4]. The proposed cut-off value (33 μg/L) was based primarily on studies of cardiac arrest patients not treated with therapeutic hypothermia [5–9]. Recent studies evaluating NSE as a prognostic marker in hypothermia treated cardiac arrest patients have shown divergent results; while some studies support the proposed cut-off value [10, 11], other studies do not [12–15]. As a consequence, the use of NSE as a biomarker to predict outcome after cardiac arrest has been questioned. Little is known about the influence of hypothermia on the release-curve and turnover of NSE. Also, the influence of handling and storage of samples need to be further investigated.

Currently, there are several commercially available NSE immunoassays from different manufacturers and no common standard. Several analytical as well as pre-analytical factors may affect the measured result [16]. For instance, based on the interference of NSE from erythrocytes it has been recommended that an index of haemolysis to be performed prior to analysing the sample [1], and this is not routinely done. Also, determination of NSE was performed on fresh samples in some studies [6, 17] and retrospectively using frozen samples in other [13, 18, 19], which may have affected the results. Ramont et al. found no effect on NSE-levels by the storing of serum for up to 9 months [1], but the effect of long term storing has not been investigated. In addition, methodological differences, such as differences in antibody binding-site and/or specificity and calibration errors may affect the measured levels of NSE [16]. Since methodological differences and analytical errors may explain some of the discrepancies regarding NSE results, several authors have advised against the use of the previously specified cut-off level of NSE for determination of poor prognosis after cardiac arrest [20–22].

In order to assess the effect of long term storing, we analysed NSE in serum on original fresh samples, and 4–7 years later on frozen and stored samples collected simultaneously. We also compared two automated immuno-assays for NSE from two different manufacturers, using both fresh and long term frozen samples.

## Methods

This study was performed at Skane University Hospital, Lund, Sweden, on samples collected from hypothermia treated cardiac arrest patients in the intensive care unit between November 2003 and December 2006. The study was approved by the Regional Ethical Review Board at Lund University (411/2004), and informed consent was sought from next of kin or, retrospectively, from the patient. The method comparison on fresh venous samples utilized anonymized patient material, and consent was waived.

### Study samples

Parallel samples from 51 patients with a mean age 63.8 +/−16,0 years, were analysed. Thirty-three of the patients (65%) were male. All patients were treated with therapeutic hypothermia at 33°C for 24 hours after cardiac arrest with a controlled rewarming phase of 8 hours. Thirty-four patients had cardiac arrest of cardiac origin (67%). Sixteen of 51 patients (31%) remained unconscious until death and a total of 18 patients died during the 6 months follow up (35%).

Serial serum samples from hypothermia treated cardiac arrest patients were collected during the first 72 h after cardiac arrest. Two arterial blood samples from each patient were collected 48 h after the cardiac arrest. The first sample (the original sample) was centrifuged and kept refrigerated if analysed within 24 h, otherwise the sample was frozen (−20°C) and analysed within a week. The simultaneously collected second serum sample was centrifuged and frozen at −70°C for later analysis (stored sample). The stored samples were thawed once and aliquoted during storage. The samples were reanalysed after 4–7 years of storage. Patients with intraaortic balloon-pump counter-pulsations (IABP) were excluded from this study because of the risk for low-grade intravascular haemolysis. All samples with visible haemolysis were discarded on arrival at the laboratory.

In addition, 29 fresh venous serum samples were used in the study. These fresh samples were aliquoted in 2 portions and stored (less than one week) at −20°C until analysis.

### NSE analysis

All samples were analysed at the Clinical Chemistry Laboratory, Skane University Hospital, Lund, Sweden. Determination of NSE in the original samples was performed using the LIAISON®NSE, DiaSorin S.p.A, Saluggia, Italy. Detection limit and reference interval for this method was 0.04 μg/L and <18.3 μg/L respectively. The coefficient of variation (CV) for the analysis method was 7%.

The stored samples and the fresh venous samples were analysed using two different methods, the LIAISON®NSE and the NSE Cobas e601, Roche Diagnostics, Mannheim, Germany. Detection limit and reference interval for this method was 0.05 μg/L and <17 μg/L respectively. The CV for the analysis method was 4%.

The LIAISON® NSE method is a fully automated monoclonal double (sandwich) chemiluminescence immunoassay, where the serum is incubated with an antibody coated magnetic particle and a luminescence-labelled tracer. The resulting luminescence is indicative of the amount of NSE in the sample. The result is presented in μg/L. (Package insert LIAISON® NSE, DiaSorin, ref 314561).

The NSE Cobas e601 method is a fully automated electrochemiluminescence immunoassay, where the NSE in the sample reacts with a biotinylated monoclonal NSE-specific antibody and a ruthenium labelled NSE –specific monoclonal antibody. After addition of streptavidin –coupled micro particles the antigen/antibody complex is detected using chemiluminescence. The result is determined via an instrument specific calibration curve and presented in μg/L. (Package insert NSE, Roche 2010–03).

According to the manufacturers, both analyses measure the γ subunit of NSE.

### Statistics

Patient characteristics are presented as numbers and percentages. The NSE results were not normally distributed, thus, data are presented as median, interquartile range (IQR) and range.

Three comparisons of paired samples were made: first between the paired original and stored samples using the LIAISON®NSE analysis method, second between LIAISON®NSE and Cobas e601on the stored samples and third, between LIAISON®NSE and Cobas e601 on the fresh venous samples.

Bland-Altman plots were produced for the paired data of the three comparisons. Bias, limits of agreement and 95% confidence intervals were calculated. The difference between the paired samples were analysed using Students *t*-test for paired data or Wilcoxon matched sign rank test according to normality distribution.

Correlation, linearity and the equation of the line were produced for the three comparisons. GraphPad Prism 5.0 (GraphPad Software, Inc. CA, US) was used for the analyses, p < 0.05 was considered significant.

## Results

### Results for NSE in the paired original and stored samples with the LIAISON®NSE method

**Figure 1:**
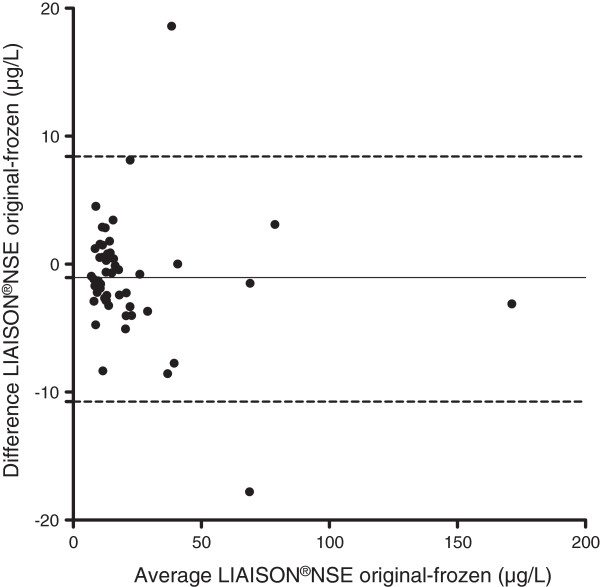
**Bland-Altman plot showing the difference in NSE between the original and stored samples, simultaneously collected from the same individual on the y-axis, versus the mean between the original and stored NSE values on the x-axis.** The solid line denotes the mean difference between the analyses and the hatched line the upper and lower 1.96 SD lines. All analyses were made using the LIAISON®NSE analysis method.

**Figure 2:**
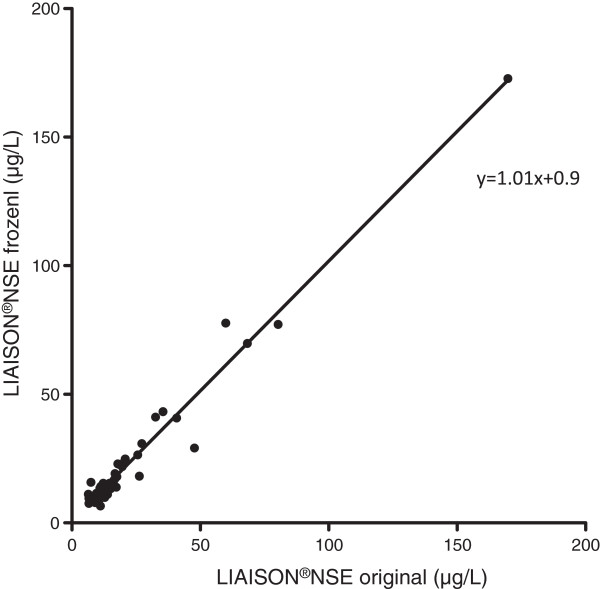
**NSE measured using LIAISON®NSE on original and stored samples with line of equity.**

The median value for NSE in the original samples according to the LIAISON®NSE method was 13.9 μg/L (IQR 10.9-20.5 μg/L, range 6.5-169.7 μg/L). The median NSE value in the stored samples was 14.3 μg/L (IQR 11.1-23.0 μg/L, range 6.5-172.8 μg/L). The differences between the paired original and stored samples were normally distributed with a mean difference of −1.2 μg/L +/−4.8 μg/L (p = 0.12). Figure 1 shows the Bland–Altman plot of the samples. NSE was higher in 34/51 (67%) stored samples compared to the original ones. The correlation coefficient was r = 0.89 (95% CI 0.80 to 0.93) (p < 0.0001), (Figure 2). The equation of the line was y = 1.01 x +0.9 with a 95% CI for the slope of 0.96-1.06, which can be translated into an increase of 1% (95% CI −4 to +6%) for stored samples over the whole range of examined values.

### Results for NSE with the LIAISON®NSE and the NSE Cobas e601 methods on stored samples

**Figure 3:**
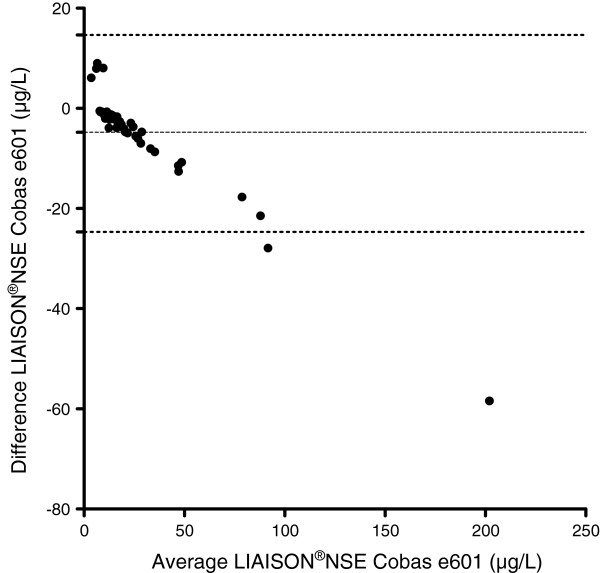
**Bland-Altman plot showing the difference between the stored samples analysed using the LIAISON and the NSE Cobas e601 respectively.** The samples were exposed to identical preanalytical conditions. The difference between the NSE levels analysed on the LIAISON®NSE and the NSE Cobas e601 are shown on the y-axis and the mean between the LIAISON®NSE and the NSE Cobas e601 NSE levels on the x-axis. The solid line denotes the mean difference between the analyses and the hatched line the upper and lower 1.96 SD lines.

**Figure 4:**
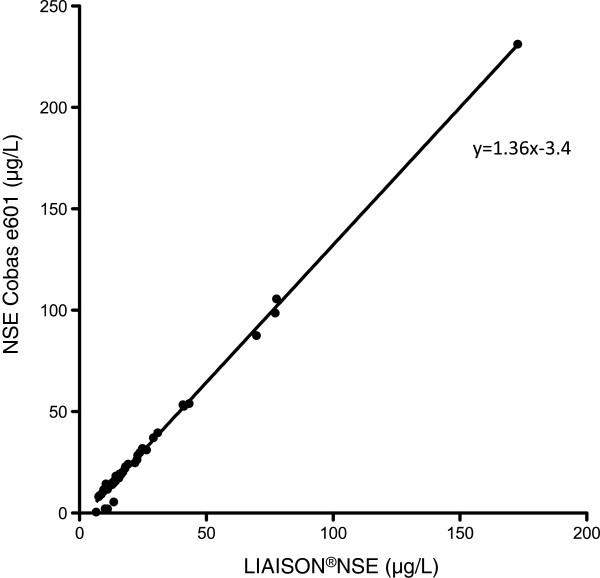
**NSE measured using stored samples and the LIAISON®NSE and the NSE Cobas e601 respectively, with line of equity.**

The median NSE-level in stored samples according to the NSE Cobas e601 method was 16.3 μg/L (IQR 11.8-28.5 μg/L, range 0.5-231.2 μg/L). The mean difference between LIAISON®NSE and the NSE Cobas e601 methods on the stored samples was −5.4 μg/L, (SD 9.5 μg/L), with 95% limits of agreement +/−19.4 μg/L (Figure 3).The differences between the paired NSE results were not normally distributed. The difference between the paired data was significant (p < 0.0001). The NSE Cobas e601 method showed consistently higher NSE values than the LIAISON®NSE method, with a higher value in 49/51 (96%) of samples. The correlation coefficient was r = 0.97 (CI 0.95-0.98, p < 0.0001). Figure 4 shows the scatter of samples and the assessed linearity. The equation of the line was y = 1.36 × −3.4 with a 95% CI for the slope of 1.33 to 1.38, which can be translated into a 36% (95% CI 33–38%) increase in NSE level using the NSE Cobas e601 method.

### Results for NSE with the LIAISON®NSE and the NSE Cobas e601 methods on fresh venous samples

**Figure 5:**
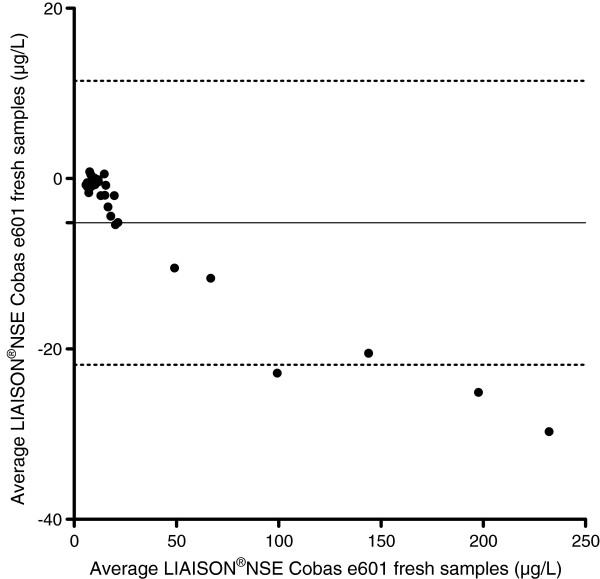
**Bland-Altman plot showing the difference between the fresh samples analysed using the LIAISON®NSE and the NSE Cobas e601 respectively.** The analysed samples were exposed to identical Preanalytical conditions. The difference between the NSE levels analysed on the LIAISON®NSE and the NSE Cobas e601 are shown on the y-axis and the mean between the LIAISON®NSE and the NSE Cobas e601 NSE levels on the x-axis. The solid line denotes the mean difference between the analyses and the hatched line the upper and lower 1.96 SD lines.

The median NSE-values for the fresh venous samples were 12.0 μg/L (IQR 8.2-18.8, range 5.4-217.3 μg/L) and 14.0 μg/L (IQR 8.5-23.5, range 6.1-247.0 μg/L) for the LIAISON®NSE- and the NSE Cobas e601 -methods respectively. The mean difference between the two methods was −5.2 μg/L, (SD 8.5 μg/L), with 95% limits of agreement +/−16.3 μg/L (Figure 5).

**Figure 6:**
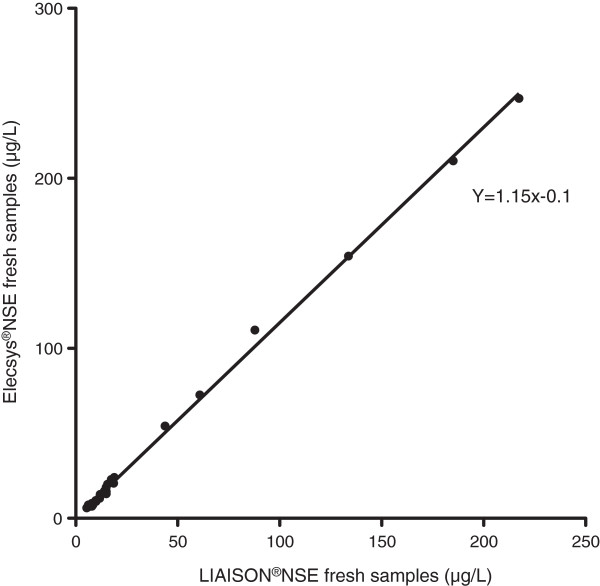
**NSE measured using fresh samples and the LIAISON®NSE and the NSE Cobas e601 respectively, with line of equity.**

The differences between the paired data for the fresh venous samples were not normally distributed. The difference between the paired data was significant (p < 0.0001). The NSE Cobas e601method showed higher NSE values than the LIAISON®NSE method in 25/29 (86%) of the samples.The correlation coefficient between was r = 0.99 (95% CI 0.97 to 0.99). The equation of the line was y = 1.15 × - 0.1, CI 1.13- 1.17, which can be translated into a 15% (95% CI 13–17%) increase in NSE level using the NSE Cobas e601 method (Figure 6).

## Discussion

The main finding of this study is that there were significant differences in NSE results between the two measuring methods, the NSE Cobas e601 consistently showing higher values than the LIAISON®NSE, on fresh (15%) as well as on stored (36%) samples.

The samples stored over a prolonged period of time (4–7 years) showed a minor NSE increase (mean 1.2 μg/L) as compared to the original samples, and a non-significant difference between the paired data. This is in keeping with the results from a previous study where samples frozen up to 9 months were investigated [1]. Despite this, the scatter of the paired results was substantial in some patients with occasional values diverging as much as 18 μg/L without a trend for any particular pattern in the scatterplot (Figures 1 and 2). Thus, long-term storage may lead to significantly altered levels of NSE on an individual sample level. This is a relevant concern since batch analysis of stored samples is sometimes used to eliminate the risk of clinicians being biased by the NSE results. Such a strategy is used in the Target Temperature Management after cardiac arrest trial (TTM-trial) [23]. As part of the trial, NSE will be evaluated as a prognostic marker after cardiac arrest, including the potential influence of temperature [24].

In the comparisons between the LIAISON®NSE and the NSE Cobas e601 methods on stored and fresh samples respectively, the correlations between the NSE results from the two methods were good. The correlation coefficients were 0.97-0.99, which was similar to the correlation coefficients noted by Stern [16]. However, the Roche method generated consistently higher results compared to the DiaSorin method in both the fresh venous samples and in the stored samples. An interesting finding was that the results produced by the two methods differed more when stored samples were analysed as compared to the fresh venous samples. We speculate that sample integrity may have been affected by the freeze-thaw-cycle and storage and that the sensitivity for decreased sample integrity may differ between the two analytical methods.

The discrepancies in results between the DiaSorin and the Roche methods are highly relevant with regards to the use of NSE to assess neurological outcome after cardiac arrest. In the AAN guidelines [4], mainly based on the PROPAC I study [5], a cut-off of 33 μg/L at 24–72 hours after cardiac arrest was considered strong evidence for a poor prognosis [4, 5]. In the PROPAC I study the Sangtec/LIAISON method was used [25]. In a recent study, the LIAISON®NSE and the CanAg®NSE were compared in two laboratories with substantial differences in results [26]. These results in combination with our findings strengthen the conclusion that a universal cut-off-value for NSE should not be used for prognostication purposes and highlights the need for standardisation. In our opinion, biomarkers including NSE cannot constitute the sole foundation for withdrawal of life supportive therapy [27]. NSE should rather be regarded as an adjunct in prognostication after cardiac arrest as part of a multimodal approach [15, 20, 28]. Although the absolute values apparently depend on the analytical method, the typical release curve of NSE after cardiac arrest is probably not affected [11, 12], and serial measurements are recommended to increase reliability in predictions.

Measurement of free haemoglobin reduces the risk of overestimating NSE-levels in samples where haemolysis occurred during sampling, transport or sample handling. On Cobas e601 the presence of haemolysis is automatically assessed in each sample, which is discarded if haemolysis exceeds a defined threshold value. In a cardiac arrest population, patients with intra-aortic balloon pumps (IABP) may have on-going low-grade haemolysis. Since the half-life of free haemoglobin is approximately 2–4 hours [29], considerably shorter than the 30 hour half-life of NSE [3], an accumulation of NSE relative to free haemoglobin may occur over time. As a consequence, the measured value of NSE may be inappropriately increased beyond the time-point where free haemoglobin is no longer detectable in the sample. This is a cause of concern when using NSE for prognostication after cardiac arrest. In support for this reasoning, high NSE-levels, unrelated to detectable brain injury was found in patients undergoing coronary surgery, where the haemolysis was caused by cardiopulmonary bypass and blood-collection devices [3].

### Limitations

This is a pilot study using a small number of samples, but still it illuminates the risk of recommending strict cut-off levels for NSE, without carefully addressing potential methodological problems and specifying the measuring method as well as sample handling and storage.

This study was not designed for prognostication purposes; rather we aimed at addressing potential confounders when using NSE as part of a prognostic model. Due to the design of the study, we consider it inappropriate to disclose sensitivity/specificity or cut-off values for a poor outcome.

In the study we have tried to reduce the effect of preanalytical error by using paired blood-samples collected by the same personnel and handled in the same way en route to the laboratory (the original and stored samples), while the method comparisons on stored and fresh samples were made on identical samples, thereby eliminating differences in sample handling. All determinations of NSE were performed at the same laboratory, to eliminate the effect of inter-laboratory variation [30]. When comparing published results using NSE as a biomarker for neuronal injury, variability in results may also include inter-laboratory variation, which was not addressed in the current study.

## Conclusions

There are two main findings in this study; first that frozen NSE samples remained relatively stable during long term storage, and second that there was a consistent and significant difference between the LIAISON®NSE and the NSE Cobas e601 method, with the latter showing consistently higher values in both fresh and stored samples. NSE should be further evaluated as a prognostic biomarker after cardiac arrest and in parallel an international standard should be defined.

## Abbreviations

AAN: American Academy of Neurology
IABP: Intra aortic balloon-pump counter-pulsations
NSE: Neuron specific enolase.
